# Application of nano and microformulations to improve the leishmanicidal response of quinoline compounds: a brief review

**DOI:** 10.3389/fchem.2025.1622566

**Published:** 2025-07-25

**Authors:** Angel H. Romero, Karina N. Gonzalez, Marcos A. Sabino

**Affiliations:** ^1^Grupo de Química Orgánica Medicinal, Facultad de Ciencias, Universidad de la República, Montevideo, Uruguay; ^2^Centro de Química, Laboratorio de Polímeros, Instituto Venezolano de Investigaciones Científicas (IVIC), Caracas, Venezuela; ^3^Unidad de Química Básica, Facultad de Farmacia, Universidad Central de Venezuela (UCV), Caracas, Venezuela; ^4^Grupo de Investigación B5IDA, Departamento de Química, Universidad Simón Bolívar, Caracas, Venezuela

**Keywords:** leishmanicidal agent, DDS, quinoline drugs, nanoparticles, leishmania, amastigote

## Abstract

The quinolines represent an important scaffold for the development of leishmanicidal agents. In particular, the use of nano and microformulations has emerged as a powerful tool to improve the therapeutic profile of leishmanicidal drugs, favoring bioavailability, transportation to key targets, metabolic protection, and immunostimulating responses. This mini-review seeks to provide a general perspective about the use of nano/microencapsulation for the development of leishmanicidal formulations based on quinoline, giving an overview of the various cases of encapsulation, analyzing the repercussions of the type of polymeric matrix (synthetic or natural polymer), type of formulation (polymeric or metallic nanoparticles, micelles, liposomes, etc.), drug loading percentage, and release rate of quinoline drug.

## 1 Introduction

Leishmaniasis is one of the six main tropical diseases by the World Health Organization (Shafiei et al., 2014), caused by protozoan kinetoplastid intracellular parasites of *Leishmania* spp. (Murray et al., 2005). The disease presents three clinical manifestations: cutaneous leishmaniasis (CL), visceral leishmaniasis (VL), and mucocutaneous leishmaniasis (MCL) (Murray et al., 2005). The disease is present in more than 95 tropical and subtropical countries with extensive morbidity and mortality, registering between 0.7 and 1.3 million new cases and 26,000 and 65,000 deaths annually (Kumar, 2021; World Health Organization, 2023).

There are no existing vaccines, and the current chemotherapies based on pentavalent antimonials (e.g., glucantime and pentostam), pentamidine, amphotericin B (AmB), and miltefosine present several limitations, including high cost, high toxicity, low therapeutic efficacy, substantial side effects (affecting the heart, liver, and kidneys), development of resistance, and prolonged treatment duration (30–60 days) (Nih.gov, 2022; Aronson et al., 2016; Kumari et al., 2022). Strategies based on combination therapies (Mota et al., 2024; Sundar et al., 2024) and repositioning drugs have been alternatively used (Chartlon et al., 2017). In the last decades, nanotechnology has provided new opportunities in the treatment of leishmaniasis through the design of drug delivery systems (DDSs) to load leishmanicidal drugs (Sun et al., 2019). These DDSs have provided a high efficacy, high target delivery effect, low toxicity, and prolonged systemic circulation lifetime (de Carvalho et al., 2013). The use of DDSs favors the bioavailability and protects the drugs from metabolic degradation (Gershkovich et al., 2009). In addition, their lipophilic nature facilitates the accumulation of the encapsulation inside the macrophage through phagocytosis (Muller et al., 2001; Kumara et al., 2014), accumulating inside the lipophilic phagolysosome, where the intracellular amastigote parasite is localized (Seguin and Descoteaux, 2016). The latter favors an effective intracellular concentration of the nanocarrier, reducing either the macrophage toxicity or the drug dosage. DDS nanocarriers could favor immunological activation of infected macrophages (Nicolete et al., 2011).

**FIGURE 1:**
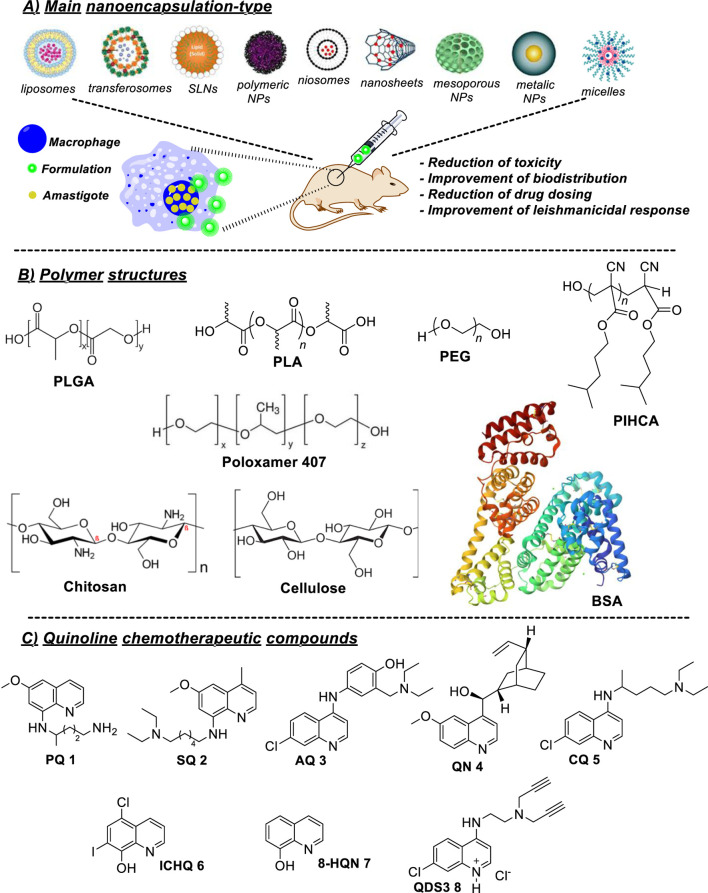
**(A)** Main type of nanosystems; **(B)** structure of synthetic (PIHCA, PLA, PEG, and PLGA), surfactant (poloxamer 407), and natural (chitosan, cellulose, and BSA) biopolymers for nanoencapsulation of leishmanicidal quinolines 1–8; and **(C)** structure of leishmanicidal quinolines used for DDS.

Against leishmaniasis models, a series of DDSs including the following: i) liposomes (mean size 100–200 nm); ii) transferosomes (mean size 100–200 nm); iii) solid lipid nanoparticles (SLNs) (mean size 10–1000 nm); (iv) polymeric nanoparticles (NPs) (mean size 10–1000 nm); (v) niosomes (mean size 10–100 nm); (vi) carbon nanosheets/nanotubes (mean size 0.4–30 nm); (viii) mesoporous silica NPs (mean size 30–300 nm); (ix) metallic NPs (mean size 20–150 nm); (x) micelles (Figure 1A) have been employed for the construction of DDSs coated with leishmanicidal drugs (Akbaria et al., 2017; Sun et al., 2019; Jamshaid et al., 2021; Maza Vega et al., 2023; Prasanna et al., 2021; Gonzalez et al., 2024). NPs or MPs, multi-lamellar vesicles, niosomes, liposomes, and microspheres have been used as DDS models for intracellular accumulation. Against VL, liposomes (Rathore et al., 2011) and iron oxide NPs (Kumar et al., 2017) have improved the bioavailability, target delivery, and therapeutic effect of the amphotericin compared with the free drug. Other typical DDS, mainly used against the CL model, are the polymeric NPs and MPs (de Carvalho et al., 2013; Want et al., 2014). These last types of DDSs are mainly based on synthetic [poly lactic-co glycolic acid (PLGA), poly lactic acid (PLA), poly ethylene-glycol (PEG), and polyisohexylcyanoacrylate (PIHCA)], as well as on natural polymers [chitosan, cellulose, or bovine serum albumin (BSA)] (Figure 1B) (Manoochehri et al., 2013; Esfandiari et al., 2019; Akbaria et al., 2017; Prasanna et al., 2021; Gaspar et al., 1992a).

As a coated drug, the quinoline represents a privileged scaffold to design leishmanicidal agent by its association with aspects crucial to *Leishmania* survival such as dysfunction of parasite mitochondria, accumulation into the macrophage phagolysosome, and macrophage immunostimulation (Romero and Delgado, 2025; Romero et al., 2019; Del Carpio et al., 2025; Yaluf et al., 2025). In addition, from a synthetic point of view, the quinolines, in particular, the 4-aminoquinolines, are structurally versatile and can generate a variety of functionalized quinolines (Delgado et al., 2025). Quinolinic antimalarial drugs, including primaquine (PQ), sitamaquine (SQ), amodiaquine (AQ), quinine (QN), and chloroquine (CQ), have emerged as the best candidates for reposition studies (Avanzo et al., 2025). They and other quinolines like clioquinol (ICHQ), 8-hydroxyquinoline (8HQN), and a quinoline named QDS3 have been investigated for the construction of DDSs coated with quinoline (Figure 1C). Then, this mini-review aims to provide a general overview of the use of DDSs to encapsulate leishmanicidal quinolines, informing the audience about their benefits to improve the pharmacokinetic, intracellular accumulation, and *in vivo*/*in vitro* efficacy against *Leishmania* models.

## 2 Encapsulation based on polymeric NPs

One of the first examples of polymeric NPs coated with quinoline drugs for evaluation against the *Leishmania* model was developed by Gaspar et al. (1992b). They prepared a series of NPs based on PIHCA to encapsulate PQ. The PQ-loaded PIHCA NPs (PQ-PIHCA NPs) and unloaded PIHCA NPs were prepared from a formulation of colloidal suspension under acid (*pH* 3.0), followed by slow solvent evaporation (Gaspar et al., 1991). The particle size of PQ-PIHCA NPs was between 200 nm and 500 nm, with an encapsulation percentage of PQ of approximately 80%–87%. The PQ was rapidly released from PQ-PIHCA NPs at 40% at 4 h under an HIFCS environment and 20% under a physiological *pH* 7.4. Further release studies showed that the PQ was rapidly degraded in 1 h under lysosomal fraction incubation with an isohexanol release production. Biological experiments primarily showed that the PQ-PIHCA NPs were 2-fold less toxic than the free PQ for the acute toxicity model (LD_50_ values of 57.5 mg/kg vs. 33.1 mg/kg). Against an *in vitro* VL using J774G8 macrophages infected with *Leishmania donovani*, the PQ-PIHCA NPs were 20 times more potent than the free PQ (EC_50_ ∼ 0.25 μg/mL vs. EC_50_ ∼ 4 μg/mL). Interestingly, unloaded PIHCA NPs also displayed an appreciable anti-*Leishmania* response, which increased with the polymer concentration, giving an estimated EC_50_ of 6 μg/mL. An extra experiment showed that the mixture between the unloaded PIHCA NPs and the free PQ at equivalent concentrations was more active than the free PQ, which revealed the synergistic effect of the polymer in the loaded PIHCA NPs.

In 1995, PQ was encapsulated using PLA NPs to be tested against *in vivo* VL models of *L. donovani* (Rodrigues et al., 1995). The PQ-PLA NPs were prepared from a formulation of a colloidal suspension, followed by slow solvent evaporation. The association of PQ with PLA was highly dependent on *pH* and drug concentration. The efficiency of PQ encapsulation ranged from 85% to 94% at 0.5 mg/mL. The PQ-PLA NPs exhibited a unimodal size ranging between 150 nm and 200 nm with a narrow dispersion. From *in vivo* experiments, the loaded PLA NPs were well tolerated by animals under intravenous administration using a dose of 30 mg PQ bound/kg (600 mg PLA/kg) with no sign of acute toxicity. Animals treated with PQ-PLA NPs showed a significant reduction of the parasite burden, giving a lower ED_50_ dose than free PQ (ED_50_ = 6.6 mg/kg vs. 21.8 mg/kg). A similar dose of the free drug resulted in 15% weight loss in animals. The effectiveness of the PQ-PLA NPs in the suppression of amastigotes in the liver of BALB/c mice was 3.3 times more than that of the free drug.

In 2001, Heurtault and co-workers developed two PQ-loaded NPs, PQ-PLA and PQ-PLA-PEG, for testing against *in vitro* intracellular amastigotes of *L. donovani* (Heurtault et al., 2001). These were prepared by the solvent-displacement method using either PLA-PEG diblock copolymers or PLA. The NPs exhibited particle sizes between 95 nm and 316 nm and an encapsulation percentage of approximately 85%–91%. The PQ-PLA-PEG NPs showed a higher antileishmanial activity against the *L. donovani*-infected model than free PQ. Interestingly, after 2 days of incubation, PLA-PQ NPs (EC_50_ = 2.7 μg/mL) were more active than PQ-PLA-PEG NPs (EC_50_ = 4.8 μg/mL) systems and PQ (EC_50_ = 7.6 μg/mL); however, the PLA-PEG-PQ NPs (EC_50_ = 0.8 μg/mL) showed a higher activity than either free PQ (EC_50_ = 2.0 μg/mL) or the PLA-PQ NPs (EC_50_ = 1.5 μg/mL) after 5 days of incubation. Thus, the PLA was shown to be a more convenient polymeric matrix for a rapid release, whereas the PLA-PEG copolymerization was suitable for a slow release with a higher efficacy. The authors suggested that the incorporation of PEG polymer seemed to limit the contact of the NP-system with cells, which seems to be key in the delayed release of the PQ-PLA-PEG NPs.

In 2014, Kumar and co-workers prepared and proved the leishmanicidal effect of a series of NPs based on PLGA and PEG and coated with SQ (Kumara et al., 2014). The PQ-PLA NPs were prepared from a formulation of a colloidal suspension, followed by slow solvent evaporation. The SQ-PLGA-PEG NPs showed a particle size of 20–30 nm and an encapsulation percentage higher than 80%. In *in vivo* experiments infected with *L. donovani*, the SQ-PLGA-PEG NPs displayed a higher leishmanicidal effect than the free SQ, requiring a lower ED_50_ dosage than free PQ (ED_50_ = 4 mg/kg vs. 12 mg/kg). A higher inhibition of parasite load was found in the splenic tissue of hamsters treated with SQ-PLGA-PEG NPs compared to hamsters treated with the free SQ.

Beyond the synthetic polymers, the organic natural polymers have demonstrated a great applicability for the construction of DDSs coated with quinolines. Among the first examples, the Nettey group reported the use of BSA for encapsulation of AQ (Nettey et al., 2017). The AQ-BSA MPs were obtained under a spray-dryer method. They exhibited a size range between 1.9 µm and 10 µm with an average zeta potential of −25.5 mV and a drug loading average of 18.27% from an encapsulation efficiency of 91.35%. The pharmacokinetic profile of the AQ, in terms of C_max_ (4.98 μg/mL vs. 0.99 μg/mL), AUC_0→48_ (59.93 μg h.mL-1 for AQ-BSA MPs vs. 26.42 μg h.mL-1 for free AQ), and *t*_*1/2*_ (15.8 h for AQ-BSA MPs vs. 65.90 h for free AQ), was significantly improved with the use of the BSA MP system. The BSA MPs provided an acceptable release percentage of the AQ, giving 12% release after the first 30 min and 93% after 24 h. In addition, the AQ-BSA MP showed a higher bioavailability than free AQ. From the *in vitro* evaluation, AQ-BSA MPs showed a similar and low toxicity on macrophages as the free AQ. Against an amastigote-infected model using *L. donovani*, the AQ-BSA MPs showed a similar leishmanicidal response to the amphotericin B but higher than free AQ. Regarding the *in vivo* experiment, no differences were noted in terms of parasitemia in organs between AQ-BSA MPs, AQ, and amphotericin B. Importantly, a remarkably higher level of AQ was detected in the spleen (7,975 μg/kg) than in lungs (2,498 μg/kg), which is of great relevance against VL models.

Following the AQ, Nettey´s group encapsulated AQ using hydroxypromethylcellulose (HPMC) microparticles, and its leishmanicidal effect against VL models infected with *L. donovani* was proved (Nettey et al., 2022). Two MPs were prepared. One was coated with chitosan (AQ-HPMC-chitosan), and the other was chitosan-uncoated (AQ-HPMC). The MPs were prepared from a spray-drying method, and the AQ-HPMC-chitosan and AQ-HPMC displayed an average size of 7.87 µm and 5.73 μm, zeta potentials of 8.85 mV and −2.22 mV, drug loading of 18.3% and 19%, and entrapment efficiency of 92% and 95%, respectively. The SEM revealed that the particles uncoated with chitosan were relatively irregularly shaped and porous compared with chitosan-coated particles, which showed a more spherical aspect. From *in vivo* experiments and under oral treatment, the AQ, AQ-HPMC, and AQ-HPMC-chitosan showed a similar efficacy to the amphotericin B, which was evidenced by the absence of parasites in the spleen, blood, and liver. Oral results were comparable with those obtained from parenteral administration. As a result, the HPMC or HPMC-chitosan MPs were recommended for oral administration in the treatment of VL in animals, conserving the efficacy without side effects.

In 2020, Nettey´s group encapsulated quinine sulfate using BSA MPs (Allotey-Babington et al., 2020). The MPs were prepared from a spray-drying method, and the QN-BSA MPs had an average size between 2.0 µm and 5.0 µm, a polydispersity index (PDI) of 0.31, and porous particles with an irregular shape. MPs showed a zeta potential of −35 mV, and the QuN was encapsulated with an encapsulation efficiency of 95% and a drug loading of 18.9%. Into the BSA MPs, QN was released at 40% and 91% after 1 h and 24 h, respectively. The pharmacokinetic parameters were significantly improved, giving a blood concentration (*C*_max_) about 2-fold higher than free QN as well as a total drug exposure (*AUC*) that was approximately 3-fold greater than the free QN. *In vitro* experiments against the amastigote of *L. donovani* showed that the encapsulation significantly improved the leishmanicidal response compared with the free QN (40% vs. 60% of parasite burden reduction) and was slightly better than amphotericin B (56% of parasite load reduction). Against *in vivo* models of rats infected with *L. donovani*, a highly significant reduction of parasite burden was found in all key organs (blood, spleen, and liver) under QN-BSA MP treatment, which was higher than those responses derived from free QN and amphotericin B.

Recently, QN was also successfully encapsulated using HPMC and chitosan and was evaluated against *in vitro* and *in vivo* VL models (Allotey-Babington et al., 2024). The QN-HPMC-chitosan MPs and QN-HPMC MPs were prepared from a spray-drying method with an encapsulation efficiency of 93%, obtaining a similar particle size to AQ-HPMC MPs, with an average size of 6.5 µm and 7.7 µm for uncoated and coated formulations, respectively. QN-HPMC-chitosan and QN-HPMC displayed zeta potentials of 13.3 mV and −0.19 mV, respectively. The formulation showed a release percentage of QN of approximately 12% and 96% after 30 min and 24 h, respectively. From *in vivo* experiments infected with *L. donovani* under oral administration (2 weeks of treatment), either QN-HPMC-chitosan or QN-HPMC MPs showed a significant reduction of parasite burden in spleen and liver organs and blood, which were appreciably better than the leishmanicidal responses derived from mice treated with pentamidine and amphotericin B.

As a last case, Gonzaga-Franco and co-workers prepared a CQ hydrogel as a topical treatment for tegumentary leishmaniasis. More than 60% of the CQ in the formulation was released after 24 h. The formulation showed a permeation rate of 15% during the first 12 h (Gonzaga-Branco et al., 2025). In an *in vivo* study using a CL model of *L. amazonensis* with BALB/c mice for a 15-day treatment, the treatment group showed a significant reduction in parasite load and lesion size with minimal side effects using the topical formulation. Further experiments showed that the CQ-hydrogel formulation was an effective treatment for CL with a good permeation rate under the topical formulation, which was studied from an *ex vivo* skin permeation model using *Susscrofa domesticus* skin.

## 3 Liposomes and micelles

In 2015, Loiseau and co-workers reported a liposomal formulation to load the natural 2-*n*-propylquinoline for intravenous administration (Balaraman et al., 2015; Loiseau et al., 2022). The formulation showed a particle size of 160 nm, an encapsulation efficiency of the quinoline of 53%, and a quinoline load of 5% of total formulation content. From *in vitro* leishmanicidal activity against macrophages infected with *L. donovani*, the liposomal formulation significantly improved the leishmanicidal response of quinoline, giving IC_50_ values of 3.1 *µ*M and 5.8 *µ*M against axenic and intracellular amastigotes, which were significantly more active than free 2-propylquinoline (IC_50_ > 100 µM) and similarly active to amphotericin B. The liposomal quinoline formulation (CC_50_ = 74.1 µM) was shown to be less toxic than amphotericin B (CC_50_ = 58.3 µM). Against *in vivo* models of BALB/c mice infected with *L. donovani*, parasite burden reductions of about 84%, 33%, and 5% were found after 3 mg/kg, 1.5 mg/kg, and 0.75 mg/kg intravenous dosage for 5 consecutive days. The *in vivo* efficacy is in a similar range to amphotericin B (88%) under 1 mg/kg dosage administration, which revealed the potential of the liposomal formulation to enhance the leishmanicidal efficacy of quinoline drugs.

In 2016, Dos Reis Lage and co-workers prepared a DDS based on micelles using P407 poloxamer (18% w/w) to load 8-hydroxyquinoline (8HQN) (Dos Reis Lage et al., 2016). The micelles were prepared from a colloidal suspension, followed by slow solvent evaporation using dichloromethane. Against *in vitro* promastigotes of *L. amazonensis*, the micelle 8HQN (8HQN-M) showed an IC_50_ value of 0.8 μg/mL, which is comparable with the amphotericin B activity (IC_50_ = 1.0 μg/mL) and higher than free quinoline (IC_50_ = 10.6 μg/mL). 8HQN-M was barely more toxic than the free quinoline (CC_50_ values of 18.3 μg/mL vs. 31.5 μg/mL) and less toxic than the amphotericin B (CC_50_ = 7.6 μg/mL). Against the infected *in vitro* model, 8-HQN reduced the infection to 30% and 12% under 6.2 μg/mL and 50.0 μg/mL treatment, which is higher than the free quinoline that reduced the infection to 90% and 79% under the same concentrations. Meanwhile, amphotericin B decreased infection to 26% and 2% under 2.5 μg/mL and 10 μg/mL dosages, respectively. In an *in vivo* experiment using *L. amazonensis*-infected BALB/c mice, in general, the 8HQN-M significantly decreased the lesion size diameter and parasite load in the liver, spleen, and draining lymph nodes compared with free quinoline and non-incorporated micelle (B-8-HQN/M). In addition, these animals also showed significantly higher levels of IFN-γ and IL-12 cytokines as well as an increase in nitric oxide under 8HQN-M and free 8HQN, which were higher than the control group under saline treatment.

In 2020, Tavares and co-workers prepared a clioquinol (ICHQ)-containing pluronic F127 polymeric micelle system (ICHQ-M) to be tested against a *Leishmania infantum*-infected BALB/c murine model (Tavares et al., 2020). The micelles were prepared from a colloidal suspension, followed by slow solvent evaporation using dichloromethane as solvent. In general, ICHQ-M reduced the parasite load in the spleen at 15 days with barely any improvement compared with free ICHQ and 2-fold more than the miltefosine drug. Further studies showed that the treatment using miltefosine, ICHQ, or ICHQ/Mic induced significantly higher anti-parasite IFN-γ, IL-12, GM-CSF, nitrite, and IgG2a isotype antibody levels.

As a last example, Ribeiro Antinarelli and co-workers prepared a poloxamer 407-based polymeric micelles to load the *N*-(2-((7-chloroquinolin-4-yl) amino) ethyl)-*N*-(prop-2-yn-1-yl) prop-2-yn-1-aminium chloride (QDS3) for *in vitro* and *in vivo* evaluation against *L. infantum* models (Ribeiro Antinarelli et al., 2022)*.* The micelles were prepared from a colloidal suspension, followed by slow solvent evaporation using dichloromethane as the solvent. The QDS3/M showed a mean particle size of 98 nm, a negative zeta potential of −9.02 mV, and, from TEM, the nanosystem showed homogeneous spherical shapes and smooth surfaces with a PDI of 0.19. Against an axenic amastigote, the QDS3/M were 2-fold more active than the free QDS3 (IC_50_ values of 25 μM vs. 10 μM), whereas miltefosine showed an IC_50_ value of 1.9 μM. The cytotoxicity of QDS3-M and free QDS3 displayed similar CC_50_ values of 344.5 μM and 289.6 μM, respectively, which was better than the miltefosine cytotoxicity (CC_50_ = 19.8 μM). Against an infected macrophage model, QDS3-M displayed a barely higher leishmanicidal response than the free QDS3, giving reduction of infection in 78% and 68% under 10 µM treatment, respectively, whereas miltefosine showed a reduction of infection in 57% under a 5 µM dosage. From the *in vivo* experiment, a significant parasite burden reduction was found under QDS3-M, which was barely higher than the free QDS3 and clearly higher than miltefosine treatment. Finally, further studies showed that either the QDS3-M or the QDS3 displayed a significant increase in levels of IL-12, IFN-γ, GM-CSF, and nitrite compared with the negative controls.

## 4 Conclusion

In summary, DDSs have contributed to improving the pharmacokinetic, bioavailability, therapeutic, and immunological activation of quinoline leishmanicidal drugs (*e.g.*, PQ, AQ, CQ, SQ, QN, 8HQN, ICHQ, QDS3, and 2-propylquinoline) (Table 1). From the reported examples, it is clear that most of the reported DDSs to load leishmanicidal quinolines are based on polymeric NPs (eight examples), followed by examples of liposomes (2), micelles (2), and hydrogels (1). Within the NPs based on synthetic polymers, although the data are heterogeneous (difference in particle size, in polymeric matrix and type of VL model), it should be noted that the PIHCA polymer is highly convenient for improving the therapeutic efficacy of PQ against *in vitro* models and supported increasing the antiamastigote response by 20-fold compared with the free PQ. Other polymeric matrices, such as PLA or PLA-PEG, increased the leishmanicidal response by 3-fold and 1.5-fold compared with the free PQ, respectively. The remarkable leishmanicidal effect promoted by the PHICA polymer could be associated with its ability: i) as an immunostimulant to immunologically activate the infected macrophage (Gaspar et al., 1992a) or ii) as a leishmanicidal agent. Meanwhile, when the *in vivo* leishmanicidal response was analyzed, it should be noted that the PLA slightly increased the leishmanicidal response of the quinoline drug compared to the PLGA-PEG copolymer. Importantly, the copolymerization with PEG could be important for slow release, as was described for PLA-PEG-PQ systems. As a last point, it seems that the particle size has a detrimental effect, although further studies are needed to clarify this point.

**TABLE 1:** Summary of examples of drug delivery nanosystems to load leishmanicidal quinolines.

Nanosystem-type	Polymer/loaded quinoline	Leishmania strain	Experiments	Major results	References
Polymeric NPs	PIHCA-PQ (200–500 nm)	*L. donovani*	*In vitro* infected macrophage	EC_50_∼4 μg/mL (PQ) EC_50_∼0.25 μg/mL (PQ-PIHCA NPs)	Gaspar et al. (1992a)
	PLA-PQ (150–200 nm)	*L. donovani*	*In vivo* model	ED_50_ = 21.8 mg/kg (PQ) ED_50_ = 6.6 mg/kg (PLA-PQ)	Rodrigues et al. (1995)
	PLA-PEG-PQ and PLA-PQ (95–316 nm)	*L. donovani*	*In vitro* infected macrophage	EC_50_ = 7.6 μg/mL (PQ) EC_50_ = 2.7 μg/mL (PLA-PQ) EC_50_ = 4.8 μg/mL (PQ-PLA-PEG)	Heurtault et al. (2001)
	PLGA-PEG-SQ (20–30 nm)	*L. donovani*	*In vivo* model	ED_50_ = 12 mg/kg (SQ) ED_50_ = 4 mg/kg (PLGA-PEG-SQ)	Kumara et al. (2014)
	BSA-AQ (1.9–10 µm)	*L. donovani*	*In vitro* and *in vivo* models	- Improvement of pharmacokinetic parameters by using BSA-AQ - Remarkable accumulation in the spleen and lungs from BSA-AQ - Similar results between BSA-AQ and AQ	Nettey et al. (2017)
	HPMC-chitosan-AQ (5.7–7.8 µm)	*L. donovani*	*In vivo* model	- Similar results from oral and parental administration - Similar results between HPMC-chitosan-AQ and liposomal amphotericin B (AmB)	Nettey et al. (2022)
	BSA-QN (2.0–5.0 µm)	*L. donovani*	*In vivo* model	-Improvement of pharmacokinetic parameters by using BSA-QN -BSA-QN was more potent than free QN and AmB	Allotey-Babingto et al. (2020)
	HPMC-chitosan-QN	*L. donovani*	*In vivo* model	- Similar results from oral and parental administration - Similar results between HPMC-chitosan-QN and AmB and pentamidine	Allotey-Babingto et al. (2024)
	Hydrogel-CQ	*L. amazonensis*	*In vivo* model	- Significant reduction in parasite load and lesion size, with minimal side effects - Permeation rate of 15% during the first 12 h, with a slight increase to 20.3% for day 42	Gonzaga-Branco et al. (2025)
Liposome	Liposomal-2-propylquinoline (160 nm)	*L. donovani*	*In vivo* model	ED_50_ = 3 mg/kg (L-PrQ) (84%) ED_50_ = 1 mg/kg (AMB) (88%)	Balaraman et al. (2015)
	Poloxamer 407 8HQN-M	*L. amazonensis*	*In vitro and* *in vivo* models	- Provide leishmanicidal response from no active free 8HQN in an *in vitro* model - Improve *in vivo* efficacy compared with free 8HQN and AmB	Dos Reis Lage et al. (2016)
Micelles	Pluronic F127 polymer, ICHQ-M	*L. infantum*	*In vivo* model	- ICHQ was 2-fold more efficient than miltefosine - High level of IL-12, IFN-γ, and NO	Tavares et al. (2020)
	Poloxamer 407 QDS3-M	*L. infantum*	*In vitro* and *in vivo* models	- Slight difference in the *in vivo* efficacy between QDS3-M and free QDS3 - High level of IL-12, IFN-γ, and NO	Ribeiro Antinarelli et al. (2022)

Regarding the use of natural polymers, it should be noted that natural BSA, chitosan, and HPMC polymers favored the pharmacokinetics, bioavailability for oral administration, and the therapeutic efficacy of quinoline drugs (AQ or QN) compared with the free drugs, giving an *in vivo* response in same range as amphotericin B or pentamidine references for VL models. Thus, against VL models, the use of natural polymers (BSA, chitosan, or HPMC) is highly convenient for improving the pharmacokinetics and bioavailability of quinoline leishmanicidal drugs, whereas the use of synthetic polymers is highly attractive for improving the therapeutic efficacy. PHICA is able to significantly enhance the leishmanicidal effect as a consequence of an immunological activation of infected macrophages (Gaspar et al., 1992a). Finally, liposomes have been shown to be highly versatile, increasing the leishmanicidal response for both *in vitro* and *in vivo* models of CL and VL. Hydrogels, based on a unique example, are convenient for topical application against an *in vivo* CL model with a good permeation rate.

The future of encapsulating leishmanicidal quinolines in NPs and/or MPs promises to revolutionize the treatment of leishmaniasis in the pharmaceutical industry. As research advances, it is expected that the optimization of formulations based on synthetic and natural polymers will lead to more effective and safer therapies. The ability to adjust release characteristics, as observed with PLGA-PEG or PHICA systems, and the use of natural polymers like chitosan or micelles and liposomes will allow for personalized treatments tailored to different patient profiles. Furthermore, the growing interest in innovative formulations opens the door to new collaborations between researchers and pharmaceutical companies, which could accelerate the development of effective solutions against neglected tropical diseases such as leishmaniasis. With a focus on ongoing research and the potential for new technologies, this line of investigation has the potential to significantly transform current therapeutic standards. Further investigation is encouraged to calibrate the real potential of the DDSs as a standardized vehicle for leishmanicidal formulations.
